# It's more than just a bond: nurses and parents are great hospital business

**DOI:** 10.3389/fped.2024.1334302

**Published:** 2024-02-14

**Authors:** Jennifer Degl, Deb Discenza, Wakako Eklund, Carole Kenner

**Affiliations:** International Neonatal Consortium (Leadership Team), Critical Path Institute, Tucson, AZ, United States

**Keywords:** NICU, parents, neonatal nursing, clinical trials, nurse—patient relationship, premature birth, preterm birth

## Abstract

A newborn's admission to the Neonatal Intensive Care Unit (NICU) can be both stress inducing and frightening for a parent or caregiver. With nursing being a constant calming presence, a trusting bond between the parents and nurses often becomes the lifeline to survive the NICU journey. This bond impacts not only the baby and family, but also promotes the institution's reputation within the community. In today's climate of healthcare professional shortages, the power of the nurses' connections to families cannot be overlooked. It is critical in all neonatal units, especially where parents are being approached to enroll their infant in clinical research.

## Introduction

Hospital Administrators, Medical Directors, and Chiefs of Neonatology hear us loud and clear: The parent and nurse bond should not be taken lightly, and should be considered a significant contributor to a family's overall feeling of patient satisfaction, thus having lasting effects on a hospital's reputation and success. In fact, this unique relationship can actually be a revenue generator as well as a clinical trial recruitment tool and for any communities where any Quality Improvement (QI) efforts are being made. Thus, we break down the relationship of the nurse and the parent into three concise areas that can contribute to the success of your Neonatal Intensive Care Unit: (1) care (2) research and (3) satisfaction scores for both patient family and for nursing professionals. Our authors include two globally respected NICU Nurse Leaders and two globally respected NICU Parent Leaders to provide specific perspectives in each area.

## Jennifer Degl, MS, school teacher, author, speaker, advocate, mother to Joy

My daughter was born a micro-preemie at 23 weeks gestation, weighing only 575 grams. Her extremely premature birth was caused by my advanced case of placenta percreta, which nearly took my life on four different occasions during my short pregnancy, and I needed over 30 units of blood to survive her delivery. I begin with this because I had already experienced an enormous amount of trauma before my daughter was born, and the first few months of her NICU admission only added to my feelings of guilt and inadequacy as a mother, which negatively affected how I bonded with my new baby. The NICU nurses made a positive impact on my ability to connect with my new baby, who I could not swaddle, hug, comfort, nor even hold for one month, due to her critical state. I could not see her for several days after her birth because of my own complications, and I was told by her physicians that she would not likely survive. It was the nurses who arranged to take me by wheelchair to see my daughter, so that I could meet her,and they knew that I needed to bond with her. Just around my daughter's one month birthday, it was apparent to her nurses that I was becoming more depressed. I was not able to hold her yet and my pumping ability was declining, and I was still not physically healed. I began to withdraw from my support circle. One particular night nurse urged me to come back very late at night when it was quiet, so that both my daughter and I could relax. I did this for several nights in a row and I began to feel better. I was able to sing to her, talk to her, and finally hold her on my chest. This nurse turned around the entire NICU experience for me, through patience and love. These are just two examples of the many ways that nurses not only care for babies, but also for parents. They encourage parental participation in care activities in gentle and non judgemental manners that allow parents to bond with their babies despite the tubes, wires and other accessories that could hinder it.

Consenting for your baby to participate in clinical research can be confusing and worrisome for parents. They may be dealing with feelings of fear, anxiety, and sadness. Making decisions about research participation can add an additional layer of stress to an already challenging situation. Parents may struggle with the uncertainty of whether the trial will improve their baby's condition or potentially harm them. Understanding the technical details of research can be challenging for parents, especially if they don't have a medical background. Because of these reasons, and others, parents immediately look to the nurses to help them sort through the information given to them. Many parents think of the NICU nurses as an extension of themselves, both when they are present and when they are not. The parent and nurse are bonded in care of every baby in the NICU. The nurse is the person who is responsible for changing, bathing, feeding, comforting and making sure their preterm baby is safe, just as a parent would be, so it's natural that a parent would speak to their baby's nurse before consenting to research. Because of this, the NICU nurses should be made aware of, and even participate in the planning before a parent is approached to enroll their baby in research. Physicians and researchers involved in clinical trials should work closely with nurses and parents to provide clear and consise information, answer questions, and offer emotional support to both the parents and nurses of babies enrolled in research. Informed consent processes should ensure that parents have the necessary information to make a decision that is in the best interest of their baby, and the nurses should be in agreement with the research plan.

Parent satisfaction can be influenced by various factors, and it's essential to consider the unique needs and experiences of each family. While different families may prioritize different aspects of their NICU experience, several key factors tend to contribute significantly to parent satisfaction, including effective communication between all stakeholders, a family-centered approach to care, generous parent visitation policies, feeding support, appropriate patient education, and trust between the parents and nurses. Nurses act as surrogate parents, so parents must trust their nurses in order to feel comfortable leaving the bedside. Parents who trust their nurses tend to report a much higher level of patient satisfaction upon discharge, because they know that their baby was cared for by those who had both their baby's and their own best interest at heart. As a parent of a former micro preemie who spent many months in a NICU, I can tell you firsthand that the very first thing I mention (and others mention to me) when asked about my baby's NICU stay, is how much I trusted her nurses, and how they are the reason I was able to remain healthy enough to be present in my daughter's time of need and why I left the NICU with the confidence I needed to care for her after she was discharged.

## Deb Discenza, MA, mom to Becky, author, speaker, and global NICU parent leader

My daughter was born at 30 weeks gestation at 1.33243 kg in 2003 and spent 38 days in the NICU was discharged with a full team of specialists, compounded medications and oxygen and a monitor. I spent the entire NICU admission terrified that (1) someone would take my baby away from me if I cried at bedside and (2) I would hurt my daughter by simply touching her. But Nurse Donna changed all of that. She took us under her nursing wing and taught us how to care for our daughter and how to bond with her despite the equipment. Fast forward, discharge day left me stressed, worrying that I suddenly had an infant to care for not only as a parent but a lay nurse/doctor. Yet at home, I heard Nurse Donna's calm and reassuring voice in my head guiding me through each step. Nurses quite frankly become pseudo parents to not only the infant in care but the whole family as well. Honestly, they can make or break a family's short-term and long-term outcome.

And that relationship can also affect recruitment for clinical research trials. It may sound odd, but parents bringing a nurse gifts of food and cards spells one thing: Trust. Parents have no choice but to relinquish their infant's care to complete strangers. The bedside nurse has to navigate the tender emotions of the parents and other family members while alternately doing all of the early on cares essential to help the infant not only survive but thrive and go home. This trust is extremely important because while it takes time to build it, it could also be shattered in an instant. And honestly, in the moment when the child quickly becomes ill and the doctor and the nurse need consent for anything from a blood transfusion to a full-on procedure, the nurse provides reassurance in a difficult moment. So nurses must be a part of the clinical trial recruitment plan and they must review the plans and sign off before the trial can be done. Nurses know the ins and outs of their unit, the families and of course the infants. Every parent I know that has been involved in a clinical trial may have talked to the recruiter but immediately asked the opinion of their trusted partner, the NICU Nurse. To not involve a nurse through the entire process of a clinical trial is tantamount to poor recruitment. Watching trial after trial after trial fail to recruit patients, this is a huge error in part of the clinical research team.

Beyond recruitment, the NICU Nurse is a key component of the satisfaction scores of the patient family. Doctors come and go and stick around only for maybe 10 min at most with updates. The nurse is always present and there for guidance and encouragement. Nurse Donna asked us to complete the Satisfaction Survey at a kiosk in the unit and of course we scored everything as excellent. That relationship evolved into reaching into the NICU as I began my NICU Parent Leader work. And it should speak for itself that when Nurse Donna retired a number of years ago, when our daughter was a teenager, our family took her to a nice dinner to thank her for her years of service to so many families. NICU Nurses are revered, yes, but also greatly respected as a face of the care of a hospital.

## Wakako Eklund, DNP, APRN, NNP-BC, FAANP, FAAN

My nursing role gave me numerous bonding experiences both in adult critical care and the perinatal/neonatal settings. I met a family from Japan at one of the hospitals in Tennessee, whose child was given a prenatal diagnosis of anencephaly was made late in the pregnancy. I coordinated their bereavement and grief care in anticipation of delivering a child who will pass away soon after birth. I will never forget the question this father asked in Japanese. “We are delivering our daughter, only for her to pass away?” He detected a subtle Japanese regional accent when I spoke and identified the region where I was from. We were beyond surprised that his parents lived only 10 min from my parents in Japan. It was a miracle in bonding. This couple had a 34 weeker later who did well in our NICU prior to returning to Japan. We remained connected to grieve, remember the one who passed and also cherished the memories. The bond built between the nurse and a family in the midst of the crisis can be very powerful.

I experienced and recognized the gap between the researchers' needs and NICU staff's insufficient understanding about how relevant the research is in a big picture. With lack of buy in by nurses, researchers had already lost the potentially strong ally for conducting research. The trusted nurses' bond with any families can have a tremendous impact in how families would perceive the research and firm their decisions regarding the participation in the trial.

As a young cancer patient in my mid 30's, numerous researchers approached me for treatment, supportive interventions, diagnostic methods, and more. I put signatures on many consent forms willingly and often enthusiastically. I was signing for myself, and for my future patient colleagues, so to speak, and not for a fragile child of mine. This experience expanded my understanding for what the families endure when signing for their child/children. Much heavier mental, emotional and psychosocial weight is placed on parents. I did not want to be haunted by the thought that I did not do all I could to contribute to future cancer patients which may include myself. But these thoughts are not something we can ask our NICU babies' parents to think about. Not at all. In terms of satisfaction scores, I have been personally accused by parents that I did not do a good job of explaining. It was a tremendous blow, since I did care. But we nurses are dealing with numerous conditions that often emerge without sufficient notice to prepare. I do care at any hospital/facility where I have the practice privilege to work about how I impact the experience of NICU families. Unfortunately, nursing staff availability (which is out of my control) and certain any unique situations that the hospital is going through, all potentially negatively impact the family's experience. If we happen to have a family that requires more than ordinary care (psychosocial, etc) often nurses feel “drained” and feel unsupported on many occasions.

As I observed the nurses at the bedside throughout COVID and post COVID era, I am reminded of how fragile the neonatal workforce is even among the most experienced NICU is today and how worn and exhausted some of the experienced NICU staff nurses are. This resonates with me as to how easily parents are negatively impacted due to the fragility of the NICU workforce. When our neonatal workforce is weakened, this directly impacts the efforts that global initiative is putting forth, such as by the International Neonatal Consortium (INC) to promote neonatal therapy development. In many hospitals, the nurses do not feel valued or counted as valued members of the research team. Globally we are still seeing numerous hospitals where families are still not fully recognized as the essential care givers. As an international neonatal consortium, evidenced by the decision by the organization to remove the family members from the NICU, it is highly important for those at the International Neonatal Consortium to advocate that we strongly recommend family presence in NICU. It is sad to observe numerous NICUs in global settings where family presence is still limited. The environment where family may not feel welcomed or trusted as partners, will not promote a culture where parents feel the passion and motivation to support research. Trust must be mutual to bring true gains to all the parties involved. As I represent the voice of nurses nationally and globally, I join my voice with that of our parent advocates/colleagues to ensure all the stakeholders value our voice.

## Carole Kenner, PhD, RN, FAAN, FNAP, ANEF, IDFCOINN

I have been a neonatal nurse for many years-working at the bedside, conducting research, and teaching students. My focus has always been the integration of families into care. I remember what it was like for me as a new nurse in the NICU. I was scared and overwhelmed. Would I know enough to care for these fragile babies and support families? I cannot imagine what it is like for families. I always use that frame in my work. To teach families how to care for their babies, advocate for them as the care partner, and explain clinical trials their baby may be involved in the NICU stay.

Parents share fears with nurses as they are the health care professional available 24/7. Nurses often are the translator of medical terminology for the parents as they bond with us and trust us. We help the family with breastfeeding or pumping as this may be a natural process but it is not always easy especially considering the family's under tremendous stress. Most families always fear their baby may die even if we as health professionals recognize survival is possible. This constant fear erodes the family's mental health and coping abilities.

Stress and fear come to the surface when a researcher asks to enroll their baby into a clinical trial. The family often expresses to the nurses the lack of ability to hear what is being said and why the baby might benefit. The nurse tries to demystify the study, explain the informed consent, and put it in lay terms or get the researcher to more fully explain the study. Nurses work to build trust, allay fears and answer questions honestly. Nurses help boost their confidence, and act as a conduit with the rest of the healthcare and research team members.

Nurses are critical to the discharge process because this transition from hospital to home can be overwhelming. Families suddenly realize the professional support will no longer be there-at least not physically present. Families often express feelings of being overwhelmed at the thought of taking their baby home and being solely responsible for their care. Nurses ease this transition by working with families and helping build their confidence. Helping families to leave the hospital feeling well-cared for, listened to, and treated with respect is what nurses do. This trust is reflected in patient satisfaction scores which ultimately impact the hospital's reputation and community support.

The bottom line is that the institution's financial well-being resides in the reputation and patient satisfaction and ultimately translates into donor dollars. The link between satisfaction and nursing is strong. We have been the most trusted profession for many years and our partnership with parents is of paramount importance.

## Conclusion

What can you do to support these bonds beyond standing by and respecting this bond? Incorporate nurses into your organization beyond care practices and education practices. They are the eyes and ears of your NICU and they know *everything* that is going on, *seen and unseen.* And they can make the difference between your NICU beating out its competitors in your area and being the go-to for patient recruitment for clinical trials. Long gone are the days where nurses are seen and not heard. Today, they are taking on leadership roles and helping one another with mentorship, job referrals and more. Don't wait for your NICU to have been depleted of nurses before you make changes. Partner with them now, so that their skillsets are fully realized as they care for not just the patients and families, but also contribute to the growth and success of the hospital itself, and those that are at the helm.

## Data Availability

The original contributions presented in the study are included in the article/Supplementary Material, further inquiries can be directed to the corresponding author/s.

